# Vitamin B6 prevents excessive inflammation by reducing accumulation of sphingosine‐1‐phosphate in a sphingosine‐1‐phosphate lyase–dependent manner

**DOI:** 10.1111/jcmm.15917

**Published:** 2020-09-23

**Authors:** Xialin Du, Yalong Yang, Xiaoxia Zhan, Yulan Huang, Yuling Fu, Zelin Zhang, Honglin Liu, Lijie Zhang, Yanfen Li, Qian Wen, Xinying Zhou, Daming Zuo, Chaoying Zhou, Laisheng Li, Shengfeng Hu, Li Ma

**Affiliations:** ^1^ Institute of Molecular Immunology School of Laboratory Medicine and Biotechnology Southern Medical University Guangzhou China; ^2^ Department of laboratory medicine The first Affiliated Hospital Sun Yat‐sen University Guangzhou China

**Keywords:** anti‐inflammatory, inflammatory disease, sphingosine‐1‐phosphate, sphingosine‐1‐phosphate lyase, Vitamin B6

## Abstract

Vitamin B6 is necessary to maintain normal metabolism and immune response, especially the anti‐inflammatory immune response. However, the exact mechanism by which vitamin B6 plays the anti‐inflammatory role is still unclear. Here, we report a novel mechanism of preventing excessive inflammation by vitamin B6 via reduction in the accumulation of sphingosine‐1‐phosphate (S1P) in a S1P lyase (SPL)‐dependent manner in macrophages. Vitamin B6 supplementation decreased the expression of pro‐inflammatory cytokines by suppressing nuclear factor‐κB and mitogen‐activated protein kinases signalling pathways. Furthermore, vitamin B6–reduced accumulation of S1P by promoting SPL activity. The anti‐inflammatory effects of vitamin B6 were inhibited by S1P supplementation or SPL deficiency. Importantly, vitamin B6 supplementation protected mice from lethal endotoxic shock and attenuated experimental autoimmune encephalomyelitis progression. Collectively, these findings revealed a novel anti‐inflammatory mechanism of vitamin B6 and provided guidance on its clinical use.

## 1 INTRODUCTION

Inflammation is an important mechanism for the body to resist pathogen infection.^1^ However, if uncontrolled, it leads to chronic inflammation, autoimmune disease or destruction of tissues and organs, resulting in acute death.^2^, ^3^, ^4^ Macrophages are the key cells mediating innate and adaptive immunity and associated with an excessive inflammatory response.^5^ When stimulated, macrophages activate nuclear factor kappa‐B (NF‐κB) and mitogen‐activated protein kinase (MAPK) signalling pathways, releasing a variety of inflammatory factors such as interleukin‐1 beta (IL‐1β), tumour necrosis factor alpha (TNF‐α), IL‐6 and nitric oxide (NO), which cause excessive inflammation.^6^, ^7^ Meantime, a variety of strategies has been developed to prevent excessive inflammation in macrophages. It is important to clarify the potential mechanism for inhibiting excessive activation of macrophages.

Vitamin B6 is a general term for a class of vitamers related to metabolism and function.^8^, ^9^ Pyridoxal (PL), a transport form of vitamin B6, can be re‐phosphorylated by pyridoxal kinase into the active form pyridoxal 5’‐phosphate (PLP),^10^ which plays a vital role as a co‐factor in more than 150 enzymatic reactions and directly involves in metabolism and immune regulation.^11^ Vitamin B6 is considered necessary to maintain normal metabolism and immune response, especially the anti‐inflammatory immune response.^12^ A previous study reported that vitamin B6 inhibited lipopolysaccharide (LPS)‐induced expression of iNOS and COX‐2 at the mRNA and protein levels via suppressing NF‐κB activation in RAW 264.7 macrophages.^13^ It also disturbs NLRP3‐dependent caspase‐1 processing and suppresses secretion of mature IL‐1β and IL‐18.^14^ In LPS‐induced acute pneumonia, vitamin B6 down‐regulates the inflammatory gene expressions by increasing AMP‐activated protein kinase phosphorylation.^15^ In experimental sepsis, vitamin B6 reduces oxidative stress in the lungs and liver.^16^ Nevertheless, the exact mechanism of the anti‐inflammatory role of vitamin B6 is still unclear and needs further research.

Sphingosine 1‐phosphate (S1P), a potent bioactive sphingolipid metabolite, is a crucial regulator of immunity.^17^ S1P can affect the activation of NF‐κB, MAPK and other signalling pathways in many cell types, including macrophages.^18^, ^19^, ^20^ Excessive S1P levels are associated with increased inflammation and can lead to inflammatory diseases, such as inflammatory bowel disease and multiple sclerosis.^21^, ^22^ Sphingosine 1‐phosphate lyase (SPL), a PLP‐dependent enzyme, irreversibly degrades S1P into hexadecenal and phosphoethanolamine.^12^ SPL regulates the normal physiological function of the body by regulating circulating levels of S1P.^23^

In this study, a novel mechanism was demonstrated, whereby vitamin B6 prevented excessive inflammation by reducing the accumulation of S1P in a SPL‐dependent manner. S1P supplementation or SPL deficiency would significantly inhibit the anti‐inflammatory effects of vitamin B6. Furthermore, vitamin B6 supplementation prevented the development of experimental autoimmune encephalomyelitis (EAE), a mouse model of multiple sclerosis. Collectively, these findings revealed a novel anti‐inflammatory mechanism of vitamin B6 and provided guidance on its clinical use.

## 2 MATERIALS AND METHODS

### 2.1 Mice

C57BL/6 mice were from the Lab Animal Center of Southern Medicine University (Guangzhou, China). *Sgpl1^±^* mice were obtained from Jackson Laboratory, and then, these mice were bred to generate *Sgpl1* ^+/+^ and *Sgpl1* ^‐/‐^ littermates. Because homozygotes exhibit serious physical defects, such as vascular defects, polychromasia, kidney defects and palate bone fusion abnormalities, *Sgpl1* ^+/+^ and *Sgpl1* ^‐/‐^ mice were not used to carry out animal experiments. All mice were used at an age of 6‐8 weeks. All mice were maintained under specific pathogen‐free conditions in the Lab Animal Center of Southern Medicine University. All animal experiments in this study were approved by the Medical Ethics Board and the Biosafety Management Committee of Southern Medical University.

### 2.2 *In vivo* experiments

C57BL/6 mice were i.p. injected with LPS (5 mg/kg, from E. coli 0111:B4, Sigma‐Aldrich, USA) with or without vitamin B6 (Sangon Biotech, China). For vitamin B6–treated mice, the vitamin B6 dose (200 μL/mouse) was equivalent to 20 mg vitamin B6/ kg bodyweight for every day according to previous report.^14^ The vitamin B6 and LPS dissolved in 0.9% normal saline for in vivo experiments. Mice in the control group were injected with saline of the same volume with the same method. Solutions were prepared fresh immediately before injection. Serum samples were collected 24 h later. In details: 1. Take blood from the mice's eye sockets without any anticoagulant and transfer to a sterile empty tube; 2. Leave the tube in a standing position and wait 30 min; 3. Centrifuge 1500 g 10 min at 4ºC; and 4. Take out the serum for ELISA. Lethal endotoxic shock was induced in C57BL/6 mice by i.p. LPS injection (10 mg/kg).

### 2.3 Enzyme‐linked immunosorbent assay (ELISA)

IL‐1β, TNF‐α and IL‐6 levels in culture supernatant and mouse serum were measured by enzyme‐linked immunosorbent assay kit (Excell Bio, China) according to the manufacturer's protocol. In detail, dilution factors were different when serum or culture supernatant tests were performed. Serum was diluted 1:1 (serum: diluent), and culture supernatant was diluted 1:2 (serum: diluent).

### 2.4 Culture of bone marrow–derived macrophages (BMDMs)

Bone marrow cells were taken from C57BL/6J mice and placed on cell culture dishes (96 mm × 22 mm; CELLTER, China) at 37°C/5% CO_2_ in DMEM (Corning, USA) containing 10% foetal bovine serum (FBS; Corning, USA). The cells differentiated into macrophages induced by granulocyte macrophage colony‐stimulating factor (GM‐CSF, 100 ng/mL; PeproTech, USA) for 7 days. BMDMs were placed on a 12‐well cell culture plates (CELLTER) for 48 h at 37°C/5% CO_2_ in DMEM containing 10% FBS. Then, mouse macrophages were cultured with PL (500 μM, Sigma‐Aldrich, USA) for 24 h and then with LPS (from E. coli K235, 0.5 μg/ml for all in vitro stimulations) for the specified time.

### 2.5 Quantitative PCR analysis

Total RNA was purified from mouse macrophages using TRIzol reagent (Thermo Fisher Scientific, USA), and cDNA was synthesized using the First‐Strand cDNA Synthesis Kit (Thermo Fisher Scientific). Quantitative PCR (40 cycles) was performed using an Eppendorf Master Cycle RealPlex2 and a SYBR Green PCR Master Mix (Applied Biosystems, USA), and the following primers were used: m*Il1b*, 5′‐ TGGACCTTCCA GGATGAGGACA‐3′ and 5′‐GTTCATCTCGGAGCCTGTAGTG‐3′; *Tnfa*, 5′‐GGTG CCTATGTCTCAGCCTCTT‐3′ and 5′‐GCCATAGAACTGATGAGAGGGAG‐3′; *Il6*, 5′‐TACCACTTCACAAGTCGGAGGC‐3′ and 5′‐CTGCAAGTGCATCATCGTTGT TC‐3′; *Inos*, 5′‐GAGACAGGGAAGTCTGAAGCAC‐3′ and 5′‐CCAGCAGTAGTT GCTCCTCTTC‐3′; and *Actin*, 5′‐CATTGCTGACAGGATGCAGAAGG‐3′ and 5′‐T GCTGGAAGGTGGACAGTGAGG‐3′.

### 2.6 Western blotting

Macrophages were washed three times with ice‐cold PBS and lysed for 20 min on ice in RIPA buffer solution (Sigma‐Aldrich) with protease and phosphatase inhibitor cocktails (Sigma‐Aldrich). Equal amounts (20 mg) of cell lysates were resolved using 8%‐15% polyacrylamide gels transferred to PVDF membrane (Bio‐Rad, USA). Membranes were blocked in 5% non‐fat dry milk in PBS‐T and incubated overnight with their respective primary antibodies at 4°C. These respective primary antibodies' list are as follows: Phospho‐NF‐κB p65 (Ser536) (Clone: 93H1; CST, USA), NF‐κB p65 (Clone: D14E12; CST), Phospho‐p38 MAPK (Thr180/Tyr182) (Clone: D3F9; CST), p38 MAPK (Clone: D13E1; CST), Phospho‐p44/42 MAPK (Erk1/2) (Thr202/Tyr204) (Clone: D13.14.4E; CST), p44/42 MAPK (Erk1/2) (Clone: 137F5; CST), Phospho‐JNK (Thr183/Tyr185) (Clone: G9; CST), SAPK/JNK (CST), SGPL1 antibody (No. ABIN207537, 4A Biotech Co., Ltd., China) and GAPDH (Clone: D16H11; CST). The membranes were incubated at room temperature for 1 h with appropriate HRP‐conjugated secondary antibodies and were visualized with Plus‐ECL (PerkinElmer, CA) according to the manufacturer's protocol.

### 2.7 SPL enzyme activity assays

SPL enzyme activity was performed using tritium‐radiolabeled dihydrosphingosine‐1‐phosphate substrate as described previously.^24^

### 2.8 Measurement of S1P concentration

S1P levels in culture supernatant and mouse serum were measured by mouse S1P ELISA Kit (Shanghai Jianglai Industrial Limited by Share Ltd., China) according to the manufacturer's protocol.

### 2.9 EAE model

C57BL/6 mice were immunized subcutaneously with 100 mg MOG(35‐55) peptide (MEVGWYRSPFSRVVHLYRNGK) emulsified in CFA (Difco Laboratories, USA) with 500 mg *Mycobacterium tuberculosis* H37Ra on day 0. Mice also received 200 ng of pertussis toxin (Sigma, USA) by intraperitoneal injection on days 0 and 2. Classical disease score was assessed daily by assigning clinical scores according to the following ascending paralysis scale: 0, no disease; 1, tail paralysis; 2, weakness of hind limbs; 3, paralysis of hind limbs; 4, paralysis of hind limbs and severe hunched posture; and 5, moribund or death. Classical clinical scores were assigned based on ascending paralysis development. For vitamin B6–treated mice, the vitamin B6 dose (200 μL/mouse) was equivalent to 20 mg vitamin B6/ kg bodyweight for every day. Serum samples were collected at thirty days.

### 2.10 Sphingosine 1‐phosphate administration

S1P (Sigma) was solubilized in methanol (10 mM). The mice received 85 μg/kg of S1P diluted in 100 mL 0.9% normal saline‐infused under gravity into the right atrial port of the Swan‐Ganz catheter over a period of 20 min. Mice in the control group were injected with saline of the same volume with the same method.

### 2.11 Statistics

All experiments were performed at least twice. When shown, multiple samples represent biological (not technical) replicates of mice randomly sorted into each experimental group. No blinding was performed during animal experiments. Determination of statistical differences was performed using Prism 5 (GraphPad Software, Inc) using unpaired two‐tailed t tests (to compare two groups with similar variances), or one‐way ANOVA with Bonferonni's multiple comparison test (to compare more than two groups). Difference between mouse survival curves was evaluated by the log‐rank (Mantel‐Cox) test. *P* < .05 was considered significant.

## 3 RESULTS

### 3.1 Vitamin B6 inhibited pro‐inflammatory cytokine production in vivo and in vitro

**Figure 1:**
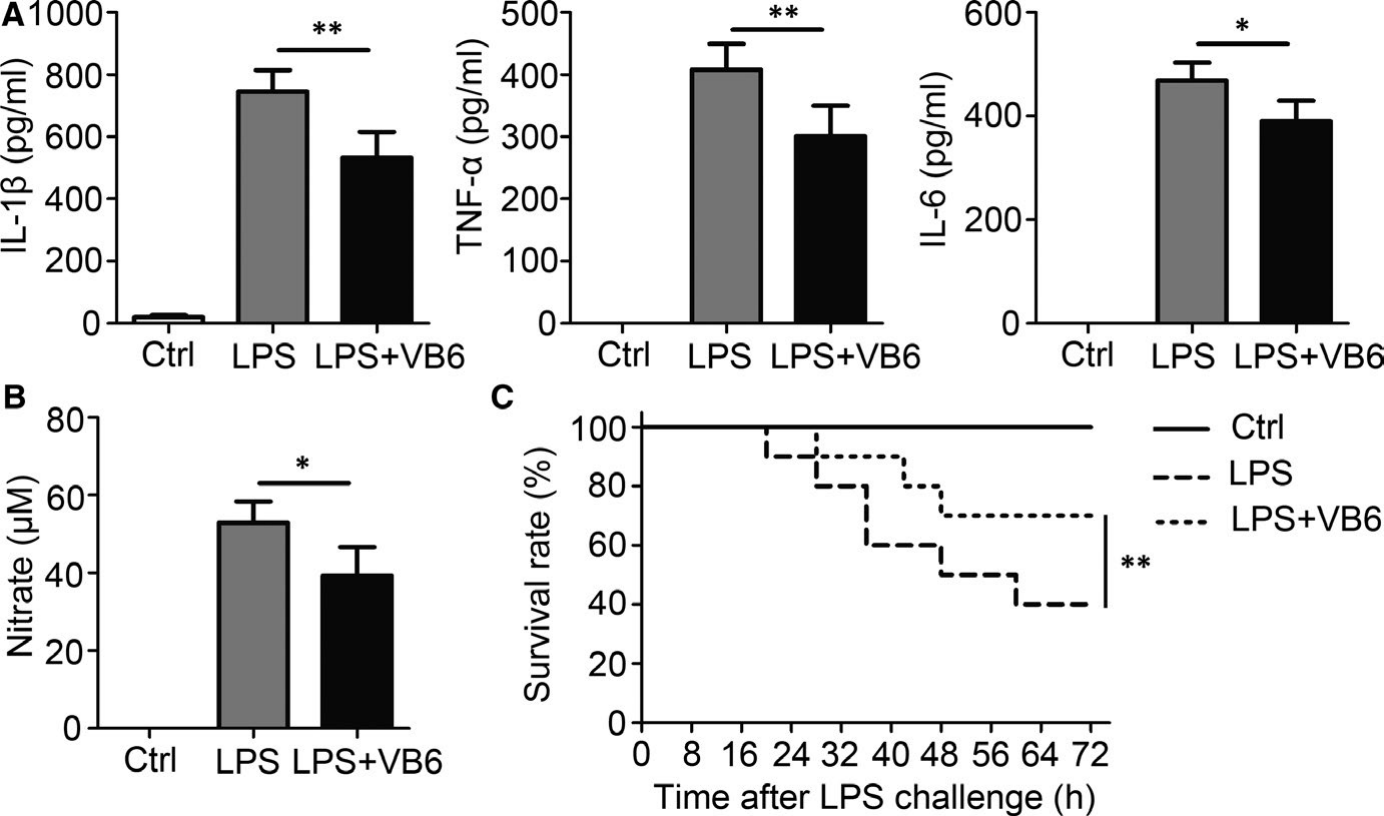
Vitamin B6 suppressed pro‐inflammatory cytokine production in vivo. (A‐B) C57BL/6 mice aged 8 weeks were orally administrated with saline (Ctrl) or vitamin B6 (20mg/kg bodyweight), and then i.p. injected with saline or LPS (10 mg/kg bodyweight) 2 h later. After 24 h, serum samples were collected (n = 5 mice for each group). (A) The IL‐1β, TNF‐α and IL‐6 concentrations were quantified by ELISA. (B) Concentrations of NO were measured by nitrate reductase assay. (C) C57BL/6 mice aged 8 weeks were orally administrated with saline or vitamin B6 (20mg/kg bodyweight), and then i.p. injected with saline or LPS (10 mg/kg bodyweight) 2 h later (n = 10 mice for each group) on day 1. Then, these mice were given saline or vitamin B6 every day like first day. The survival rate of the mice was counted. Data shown in are the mean ± SD. *P < .05, **P < .01 and ***P < .001. Data are representative of three independent experiments with similar results

Although previous reports have shown the anti‐inflammatory activity of vitamin B6, the associated mechanisms remain unclear. The anti‐inflammatory effect of vitamin B6 was first verified in vivo. Acute inflammation was induced in mice using a low dose of LPS, and serum IL‐1β, TNF‐α, and IL‐6 levels were suppressed by vitamin B6 (Figure 1A). Likewise, serum NO levels were significantly reduced in the vitamin B6–treated groups (Figure 1B). Excessive inflammation can lead to pathological damage and death. To test the anti‐inflammatory effect of vitamin B6, a high dose of LPS‐induced lethal endotoxic shock was injected in mice. The initial time of death was delayed, and the survival rate was improved in mice treated with vitamin B6 compared to control mice (Figure 1C).

**Figure 2:**
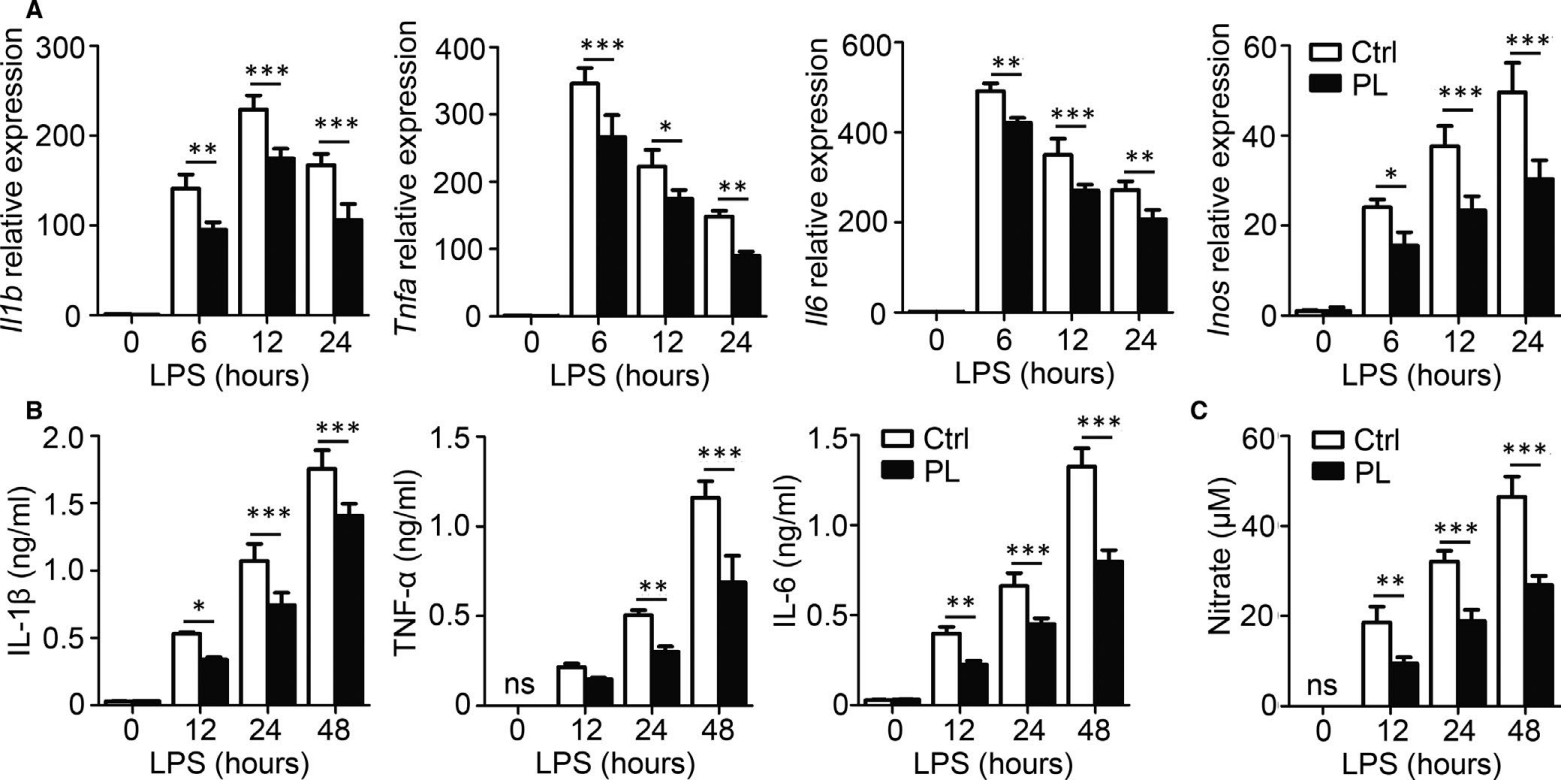
The anti‐inflammatory effect of PL in BMDMs. BMDMs were pre‐treated with PL or PBS (Ctrl) for 2 h and incubated with LPS for the specified time. (A) Expression of *Il1b*, *Tnfa*, *Il6* and *Inos* mRNA was determined by qPCR. (B) IL‐1β, TNF‐α and IL‐6 concentrations in culture supernatants for indicated time‐points were measured by ELISA. (C) Concentrations of NO were measured by nitrate reductase assay. Data shown in are the mean ± SD. *P < .05, **P < .01 and ***P < .001. Data are representative of three independent experiments with similar results

The anti‐inflammatory effect of vitamin B6 was verified in vitro. Bone marrow–derived macrophages (BMDMs) were pre‐treated with PBS or PL and then stimulated with LPS. We found that the mRNA expressions of IL‐1β, TNF‐α, IL‐6 and iNOS were reduced in the vitamin B6–pre‐treated groups compared with the control groups (Figure 2A). Moreover, BMDMs pre‐treated with PL secreted decreased amounts of IL‐1β, TNF‐α and IL‐6 (Figure 2B). The concentration of NO was reduced in the culture supernatant of BMDMs pre‐treated with PL (Figure 2C). Taken together, these results suggested a protective role of vitamin B6 in excessive inflammation.

### 3.2 Vitamin B6 inhibits pro‐inflammatory cytokines through various signalling pathways

**Figure 3:**
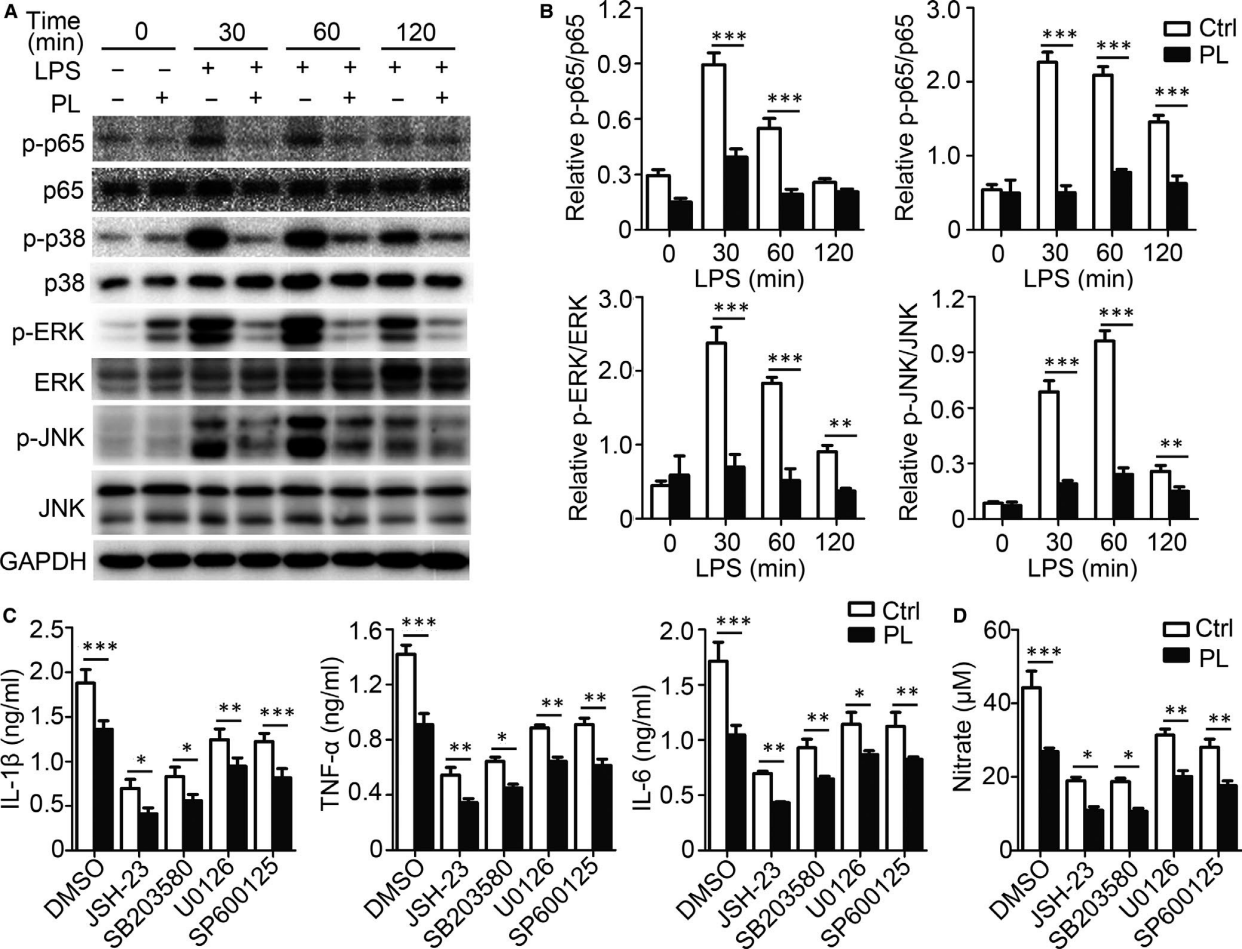
PL suppresses the expression of pro‐inflammatory cytokines through kinds of signalling pathways. (A‐B) BMDMs were pre‐treated with PL or PBS (Ctrl) for 2 h and incubated with LPS for the specified time. (A) Western blot analysis of the phosphorylation status of NF‐κB, p38, ERK and JNK. GAPDH is as an internal control. These results are from a representative experiment. (B) Densitometry quantification of band intensity of immunoblot analysis of BMDM lysates (n = 3). (C‐D) BMDMs were incubated with LPS for 24 h alone or in the presence of the NF‐κB inhibitor JSH‐23 (20 μM), p38 inhibitor SB203580 (10μM), MEK1/2 inhibitor U0126 (40 μM) or JNK inhibitor SP600125 (20 μM). The DMSO‐treated neutrophils were as control group. (C) Supernatants were collected, and IL‐1β, TNF‐α and IL‐6 concentrations were quantified by ELISA. (D) Concentrations of nitrate were measured by nitrate reductase assay. Data shown in are the mean ± SD. *P < .05, **P < .01 and ***P < .001. Data are representative of three independent experiments with similar results

The specific molecular pathways that mediate the anti‐inflammatory effect of vitamin B6 in BMDMs remain unclear. To investigate the pathways, BMDMs were pre‐treated with PL and then stimulated with LPS. PL pre‐treatment reduced the phosphorylation of p65, p38, ERK and JNK in BMDMs (Figure 3A and B). The NF‐κB inhibitor JSH‐23, MEK1/2 inhibitor U0126, p38 inhibitor SB203580 and JNK inhibitor SP600125 were used to inhibit the corresponding signalling pathway, and BMDMs pre‐treated with PL had reduced expression levels of IL‐1β, TNF‐α and IL‐6 compared with control groups if the single signalling pathway was inhibited (Figure 3C). Likewise, the concentrations of NO were reduced in the culture supernatant from BMDMs pre‐treated with PL (Figure 3D). Together, these results indicated that vitamin B6 played an anti‐inflammatory role by inhibiting NF‐κB and MAPK signalling pathways.

### 3.3 Vitamin B6 reduced accumulation of S1P by promoting SPL activity

**Figure 4:**
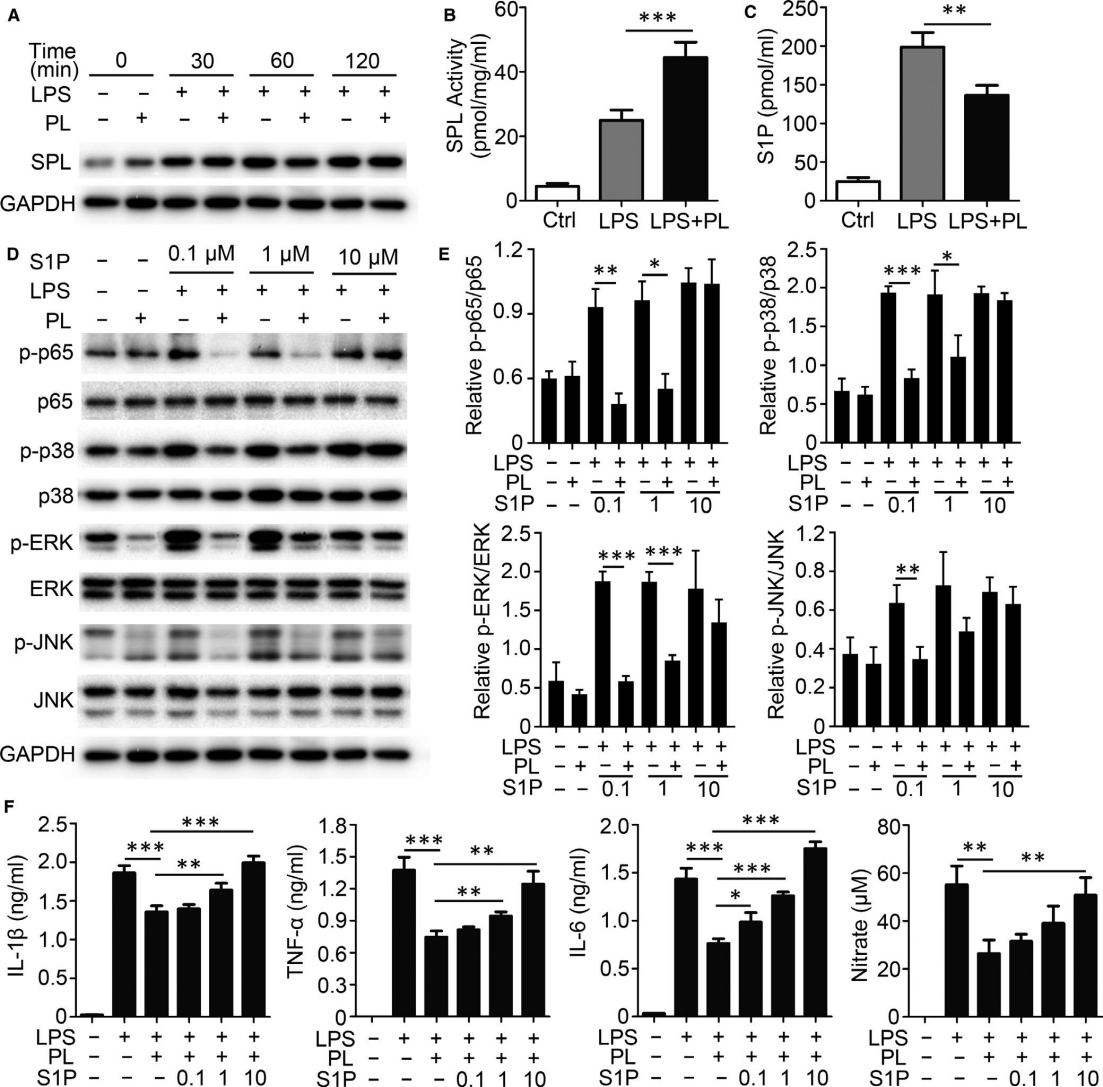
Anti‐inflammation effect of PL depends on the S1P in BMDMs. (A) BMDMs were pre‐treated with PL or PBS (Ctrl) for 2 h and incubated with LPS for the specified time. Cell homogenates were subjected to immunoblotting and probed with SPL and GAPDH antibodies. These results are from a representative experiment (n = 3) (B‐C) BMDMs were pre‐treated with PL or PBS (Ctrl) for 2 h and incubated with LPS for 24 h. (B) SPL enzyme activity levels are detected. (C) Supernatants were collected, and S1P concentrations were quantified by ELISA. (D) BMDMs were pre‐treated with PL or PBS or/and with different concentrations of S1P for 2 h, and incubated with LPS for 30 min. Western blot analysis of the phosphorylation status of NF‐κB p‐p65, p38, ERK and JNK. GAPDH is as an internal control. These results are from a representative experiment. (E) Densitometry quantification of band intensity of immunoblot analysis of BMDM lysates (n = 3). (F) BMDMs were pre‐treated with PL or PBS or/and with different concentrations of S1P for 2 h, and incubated with LPS for 24 h. Supernatants were collected, and IL‐1β, TNF‐α and IL‐6 concentrations were quantified by ELISA. Concentrations of NO were measured by nitrate reductase assay. Data shown in are the mean ± SD. *P < .05, **P < .01 and ***P < .001. Data are representative of three independent experiments with similar results

Studies on direct target molecules mediated by vitamin B6 to regulate anti‐inflammatory reactions are lacking. A previous report showed that active forms of vitamin B6 serve as a co‐factor in more than 150 enzymatic reactions.^12^ SPL, a vitamin B6–dependent enzyme, can catalyse the decomposition of S1P, which plays a vital role in regulating inflammatory signalling. Therefore, the role of vitamin B6 in negatively regulating inflammation by mediating SPL activity in macrophages was examined. Results showed that PL did not affect SPL expression in BMDMs (Figure 4A). However, SPL activity was significantly enhanced when PL was added (Figure 4B). S1P, a catalytic substrate of SPL, was significantly decreased in the PL addition groups (Figure 4C). To investigate whether PL plays an anti‐inflammatory role through the SPL‐S1P axis, S1P recovery experiments were carried out. Western blot analysis revealed that S1P supplementation significantly reduced the phosphorylation of p65, p38, ERK and JNK (Figure 4D and E). Moreover, the levels of their phosphorylation recovery were positively correlated with the concentration of S1P (Figure 4D and E). Likewise, S1P treatment lowered the ability of PL to inhibit the production of IL‐1β, TNF‐α, IL‐6 and NO (Figure 4F). A high dose of S1P completely counteracted the anti‐inflammatory effects of PL (Figure 4F). These results demonstrated that vitamin B6 played an anti‐inflammatory role by reducing accumulation of S1P by promoting SPL activity.

### 3.4 Elimination of anti‐inflammatory effects of vitamin B6 by SPL deficiency

**Figure 5:**
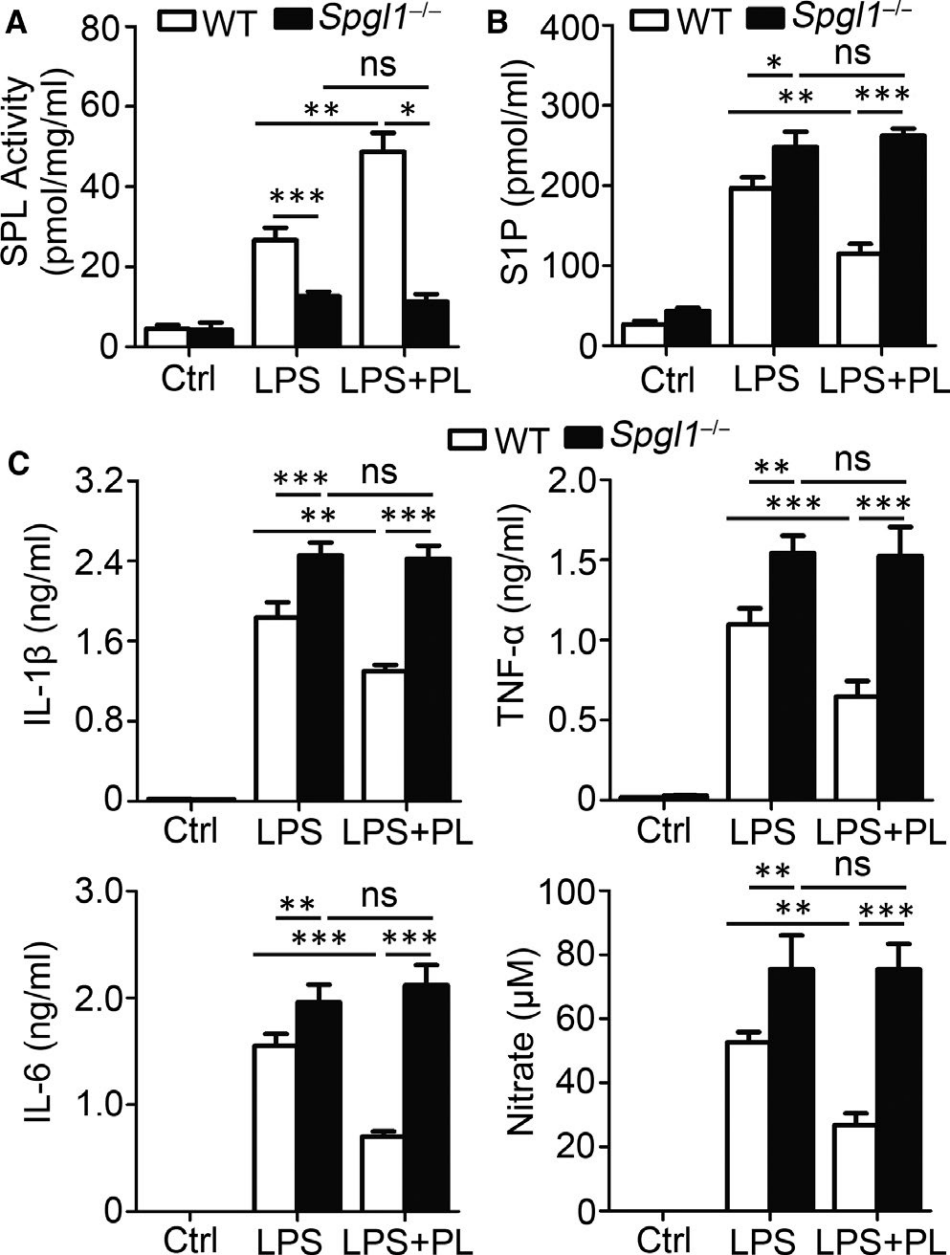
SPL deficiency limits anti‐inflammatory effect of PL. WT or *Spgl1* ^‐/‐^ BMDMs were pre‐treated with PL or PBS (Ctrl) for 2 h and incubated with LPS for 24 h. (A) SPL enzyme activity levels are detected. (B) Supernatants were collected, and S1P concentrations were quantified by ELISA. (C) Supernatants were collected, and IL‐1β, TNF‐α and IL‐6 concentrations were quantified by ELISA. Concentrations of NO were measured by nitrate reductase assay. Data shown in are the mean ± SD. *P < .05, **P < .01 and ***P < .001. Data are representative of three independent experiments with similar results

Vitamin B6 regulates anti‐inflammatory reactions through SPL acting as a direct target molecule and would not play an anti‐inflammatory role in an SPL‐deficient environment. As *Spgl1* ^‐/‐^ mice exhibit serious physical defects, such as vascular defects, polychromasia, kidney defects and palate bone fusion abnormalities, animal experiments could not be carried out. Therefore, experiments in BMDMs were carried out to investigate the effect of SPL deficiency on the anti‐inflammatory effect of vitamin B6. Results showed that SPL deficiency led to significantly reduced SPL activity (Figure 5A). Treatment with PL did not enhance SPL activity in *Spgl1* ^‐/‐^ BMDMs (Figure 5A). BMDMs from *Spgl1* ^‐/‐^ mice showed stronger expression of S1P after stimulation than BMDMs from WT mice (Figure 5B). Accumulation of S1P was not reduced in *Spgl1* ^‐/‐^ BMDMs (Figure 5B). Consistent with these results, production of IL‐1β, TNF‐α, IL‐6 and nitrate increased significantly without the influence of PL in *Spgl1* ^‐/‐^ BMDMs (Figure 5C). These results indicated the dependence of the anti‐inflammatory effect of vitamin B6 on the regulation of SPL activity.

### 3.5 S1P counteracted the anti‐inflammatory effects of vitamin B6 in vivo

**Figure 6:**
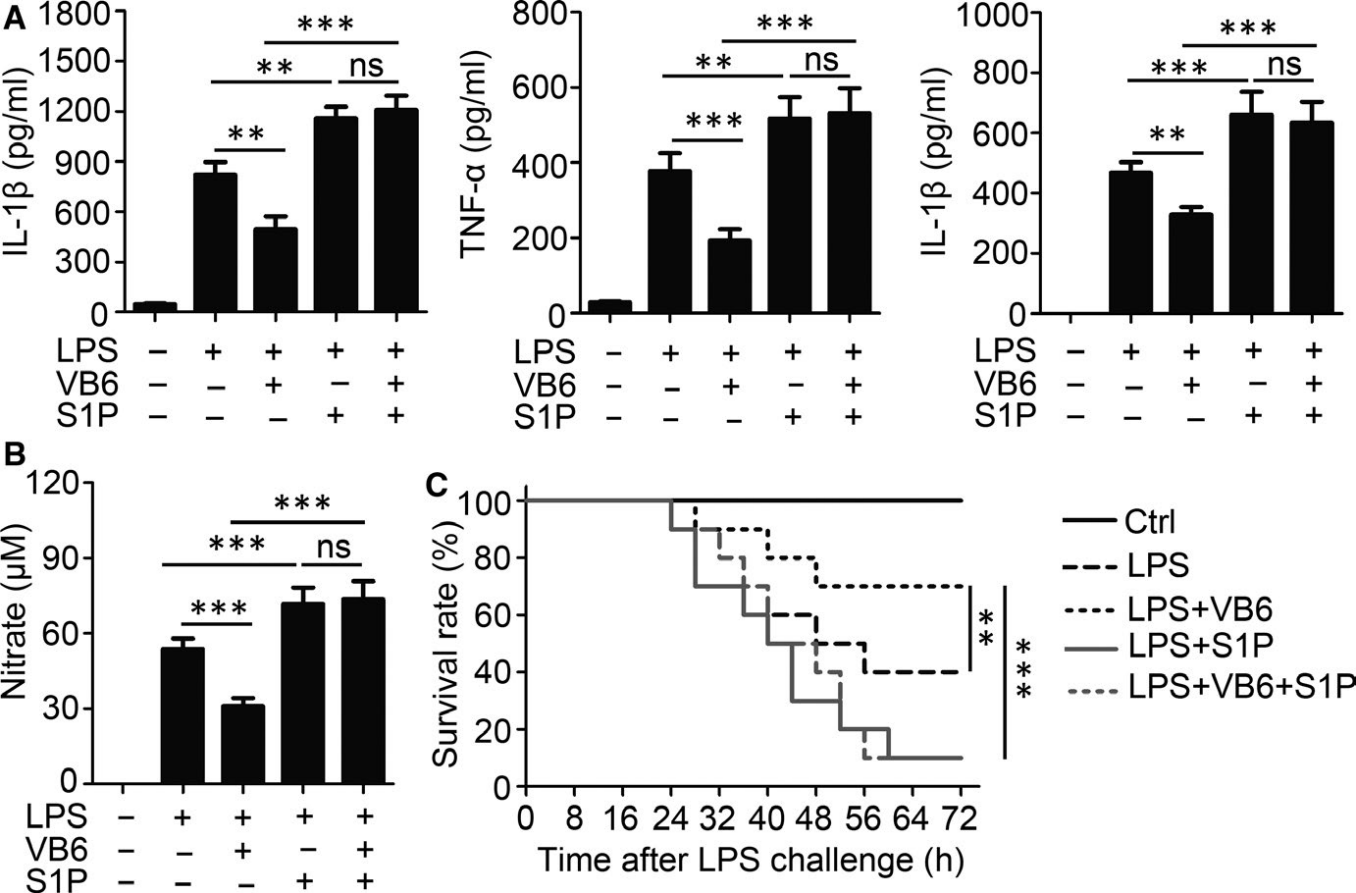
S1P reversals the anti‐inflammatory effect of vitamin B6 in vivo. (A‐B) C57BL/6 mice aged 8 weeks were orally administrated with saline (Ctrl) or vitamin B6 (20mg/kg bodyweight), i.p. injected with saline or S1P (85 μg/kg bodyweight), and then i.p. injected with saline or LPS (5 mg/kg bodyweight) 2 h later. After 24 h, serum samples were collected (n = 5 mice for each group). (A) The IL‐1β, TNF‐α and IL‐6 were quantified by ELISA. (B) Concentrations of NO were measured by nitrate reductase assay. (C) C57BL/6 mice aged 8 weeks were orally administrated with saline or vitamin B6 (20mg/kg bodyweight), i.p. injected with control solution or S1P (85 μg/kg bodyweight), and then i.p. injected with saline or LPS (10 mg/kg bodyweight) 2 h later (n = 10 mice for each group) on day one. Then, these mice were given saline or vitamin B6 or S1P every day like first day. The survival rate of the mice was counted. Data shown in are the mean ± SD. **P < .01 and ***P < .001. Data are representative of three independent experiments with similar results

In vitro assays showed that vitamin B6 suppressed inflammatory response by reducing the accumulation of S1P in a dependent manner promoting SPL activity. Thus, for the validation of the same mechanism, S1P recovery experiments were performed in vivo. Results showed that mice pre‐treated with S1P had up‐regulated expression of IL‐β, TNF‐α, IL‐6 and NO (Figure 6A, B). The anti‐inflammatory effect of vitamin B6 was completely removed by S1P supplementation (Figure 6A, B). Importantly, no differences were seen in the cytokine expressions between the S1P treatment groups and vitamin B6 and S1P co‐treatment groups (Figure 6A, B). Furthermore, we detected the survival rate of mice with lethal endotoxic shock and found that the death rate increased significantly in mice treated with S1P (Figure 6C). Vitamin B6 could not rescue the mice that were administered S1P simultaneously from lethal endotoxic shock (Figure 6C). Taken together, these results suggested that vitamin B6 played an anti‐inflammatory role by reducing the accumulation of S1P in vivo.

### 3.6 Vitamin B6 suppressed EAE progression in vivo

**Figure 7:**
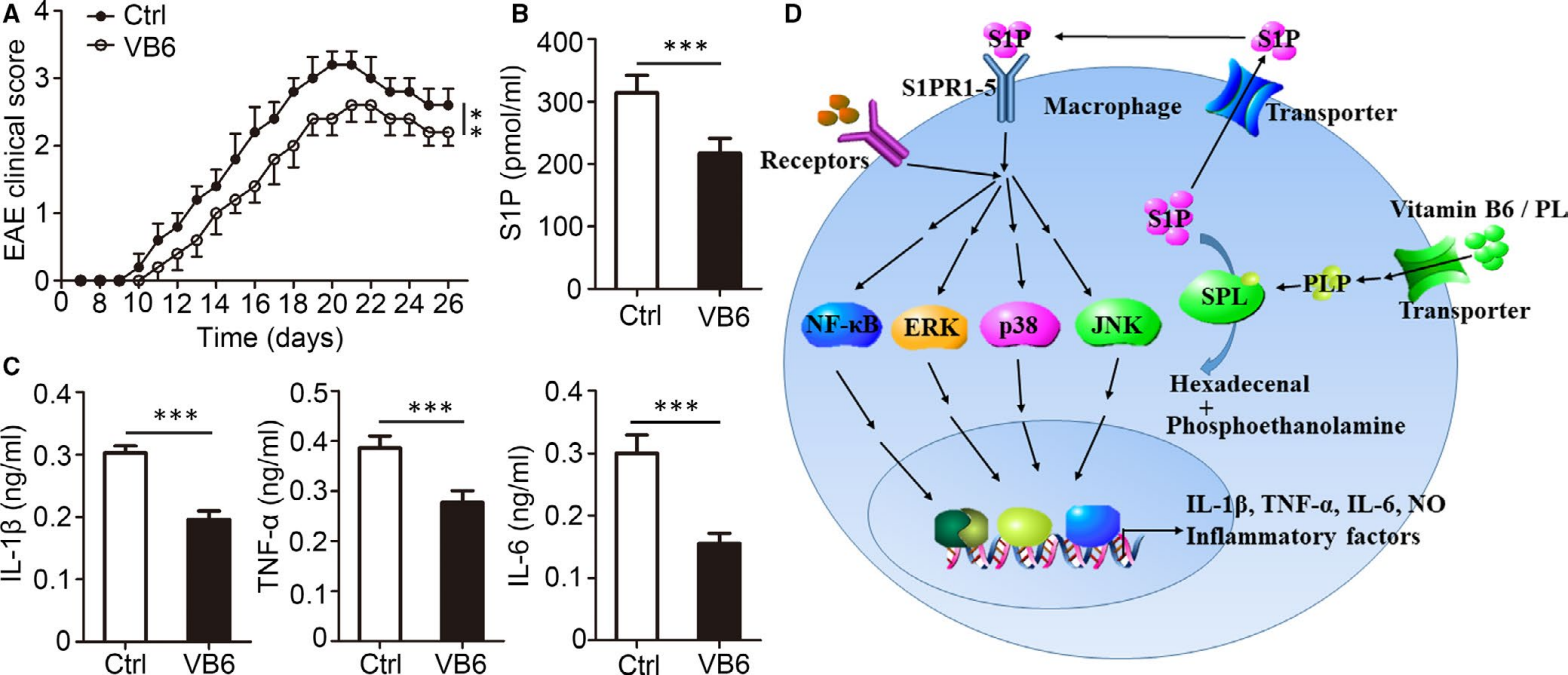
Vitamin B6 prevents EAE development. (A‐B) C57BL/6 mice aged 8 weeks were immunized with MOG (35‐55) peptide in CFA adjuvant and pertussis toxin to induce EAE. The immunized mice were orally administrated with saline or vitamin B6 (20mg/kg bodyweight) every day. (A) Mean clinical scores of EAE in immunized mice (n = 10 for each group). (B‐C) Serum samples were collected 30 days after EAE induction. (B) The S1P was quantified by ELISA (n = 5 mice for each group). (C) The IL‐1β, TNF‐α and IL‐6 were quantified by ELISA (n = 5 mice for each group). (D) A model of the role of vitamin B6 in the negative regulation of excessive inflammation in macrophage. Data shown in are the mean ± SD. ***P < .001. Data are representative of three independent experiments with similar results

Excessive inflammation is associated with the development of autoimmunity of the central nervous system. Considering the strong anti‐inflammatory properties of vitamin B6, the role of vitamin B6 in EAE was investigated. Mice were induced EAE and orally administrated with PBS or vitamin B6 daily. The EAE clinical score of the vitamin B6‐treated mice was significantly lower than that of the control groups (Figure 7A). The overall levels of S1P concentration were lower in mice treated with vitamin B6 than in control mice (Figure 7B). Similarly, ELISA detection showed down‐regulation of IL‐1β, TNF‐α and IL‐6 by vitamin B6 treatment (Figure 7C). Collectively, these findings identify that vitamin B6 prevents excessive inflammation by reducing accumulation of SIP in a SLP‐dependent manner (Figure 7D). Vitamin B6 supplementation was beneficial in controlling excessive inflammation, including the development of EAE.

## 4 DISCUSSION

Vitamins are trace organic substances required for maintaining normal physiological functions by humans and animals, including growth, metabolism and development.^25^, ^26^ The immune regulation function of vitamins has received considerable attention. The immunoregulatory mechanisms of various vitamins, such as vitamin A, C, D, B1 and B5, have been investigated.^27^, ^28^, ^29^, ^30^ In the present study, evidence was provided for the anti‐inflammatory activity of vitamin B6 in LPS‐induced acute infection and autoimmune disease. Vitamin B6 supplementation was found to reduce the accumulation of S1P by enhancing the enzyme activity of SPL.

Previous studies have shown the anti‐inflammatory activity of vitamin B6 in several inflammatory diseases. In patients with rheumatoid arthritis, vitamin B6 supplementation improved pro‐inflammatory responses by suppressing TNF‐α and IL‐6 levels.^31^ Both human and animal studies have shown vitamin B6 supplementation suppressing colon tumorigenesis.^32^, ^33^ Clinical trials found an inverse relationship between vitamin B6 intake and the risk of Parkinson's disease and Alzheimer's disease.^34^, ^35^ A new study showed that vitamin B6 supplementation effectively prevented lung inflammation.^15^ In the present study, vitamin B6 prevented toxic shock by suppressing excessive inflammation, which was consistent with the previous research.^14^ Previously, the role of vitamin B6 in multiple sclerosis was ambiguous. Animal studies in this study confirmed that vitamin B6 supplementation was beneficial to control the development of EAE. However, no blinding was performed during animal experiments. Further experiments are required to confirm these experimental results. In addition, although reports show that low plasma PLP has also been linked to rheumatoid arthritis and inflammatory bowel disease,^36^, ^37^ and other types of autoimmune diseases, it remains unclear whether vitamin B6 plays a role in these autoimmune diseases. Further research in this area could be carried out.

The anti‐inflammatory mechanism of vitamin B6 is complicated. Vitamin B6 suppresses NF‐κB activation and NLRP3‐mediated caspase‐1 activation.^13^, ^14^ Another study showed that vitamin B6 activated AMPK phosphorylation to inhibit LPS‐induced macrophage activation by activating DOK3.^15^ Consistent with these results, vitamin B6 was found to reduce the expression of pro‐inflammatory cytokines via suppression of NF‐κB and MAPK signalling pathways. However, direct target molecules mediated by vitamin B6 to suppress these signalling pathways have not been studied. Here, we demonstrated that vitamin B6 suppresses excessive inflammation by regulating SPL activity to reduce S1P levels. SPL is a PLP‐dependent enzyme and is a direct target molecule mediated by vitamin B6 to play an anti‐inflammatory role.

S1P is a bioactive sphingolipid, which binds to cell‐surface G protein–coupled receptors (GPCRs), designated S1P1‐5, and thereby mediate effects in variety of cell types, including not only macrophage but also lymphocytes.^38^ Previous reports showed that the activation of the S1P is involved in regulating differentiation of T cells, including T helper 17 and T helper 1/regulatory T cell balance.^39^ We have confirmed that vitamin B6 suppresses excessive inflammation by regulating SPL activity to reduce S1P levels in macrophages. Vitamin B6 may play a role in regulating differentiation of T cells. Further research is required to clarify this possibility.

Taken together, these findings suggest that vitamin B6 supplementation significantly suppressed excessive inflammation by directly affecting SPL activity to reduce accumulation of S1P. Thus, vitamin B6 supplementation may have important therapeutic implications in the clinical management of inflammatory diseases, such as endotoxic shock and multiple sclerosis.

## CONFLICTS OF INTEREST

The authors declare no competing financial interests.

## AUTHOR CONTRIBUTIONS

XLD, YLY, XXZ, SFH and LM: Research design; XLD, YLY, XXZ, YLH, YLF, ZLZ, HLL, LJZ and YFL: Conduction of research; XLD, XXZ, QW, XYZ, DMZ, CYZ, LSL, SFH and LM data analysis; SFH and LM: writing of the manuscript. LSL, SFH and LM: Essential reagents and materials; SFH and LM: Conduction of the experiment; and all authors: reading and approval of the final manuscript.
